# Automated high-content imaging in iPSC-derived neuronal progenitors

**DOI:** 10.1016/j.slasd.2022.12.002

**Published:** 2023-03

**Authors:** Apostolos Papandreou, Christin Luft, Serena Barral, Janos Kriston-Vizi, Manju A Kurian, Robin Ketteler

**Affiliations:** a University College London MRC Laboratory for Molecular Cell Biology, London, UK; b Developmental Neurosciences, Zayed Centre for Research into Rare Disease in Children, University College London Great Ormond Street Institute of Child Health, London, UK

**Keywords:** High-content screening, Lab automation, Neurons, Immunofluorescence, Multi-well plates, Image analysis

## Abstract

Induced pluripotent stem cells (iPSCs) have great potential as physiological disease models for human disorders where access to primary cells is difficult, such as neurons. In recent years, many protocols have been developed for the generation of iPSCs and the differentiation into specialised cell subtypes of interest. More recently, these models have been modified to allow large-scale phenotyping and high-content screening of small molecule compounds in iPSC-derived neuronal cells. Here, we describe the automated seeding of day 11 ventral midbrain progenitor cells into 96-well plates, administration of compounds, automated staining for immunofluorescence, the acquisition of images on a high-content screening platform and workflows for image analysis.

## 1 Introduction

High-Content Screening (HCS) is a powerful technique to investigate gene function in an unbiased manner within the cellular context [1], [2], [3]. Abilities to a) multiplex readouts for multi-parametric analysis and b) cell painting in combination with enhanced machine learning for image analysis have further pushed HCS to become an unrivalled technology for discoveries of cell-based phenotypes and gene/protein networks [4], [5], [6], [7], [8], [9]. This is particularly true for discoveries that aim to determine disease mechanisms or the identification of novel drug targets.

Historically, HCS has relied on easy-to-transfect cancer cell lines. More recently, a shift towards the use of primary cells and more physiologically relevant model systems has been made feasible using patient-derived cells in multi-well microplates and a two- or three-dimensional format [10,11]. For some time, the use of non-dividing neuronal cells has remained a challenge in HCS, due to the difficulties in transfection of reporter genes and the inherent variability in cell behaviour and phenotypes. The advent of induced pluripotent stem cells [12] (iPSCs) and their differentiation into a wide range of cell lineages has allowed the study of human cells from tissues that were otherwise not easily accessible, such as the brain or the heart [13]. Over the years, protocols have emerged for the use of iPSC-derived neurons in high-throughput phenotyping and high-content imaging [14], [15], [16]. However, the number of screens performed in such cell types remain low and the setup of standardized workflows for generation and high-content screening are still technically challenging.

Here, we describe a protocol for high-content screening of small molecule libraries in patient-derived neural cells and discuss potential pitfalls and challenges. We used iPSCs generated from skin fibroblasts and then applied a differentiation protocol to develop these into midbrain dopaminergic (mDA) neurons [17], [18], [19]. Day 11 ventral midbrain progenitor cells were seeded onto 96-well microplates and treated with a small molecule compound library. The cells were stained with antibodies against the autophagy marker LC3 and imaged on a PerkinElmer Opera Phenix high-content imaging platform in a semi-automated manner. Finally, an image analysis pipeline was developed to count the number of objects (autophagosomes) in these cells.

For this protocol to be implemented, fully characterized, truly pluripotent patient-derived and age-matched control iPSC lines are required. Phenotypic assay set up must take place on iPSC-derived neurons prior to the drug screen, looking for assays that distinguish patients from controls in a high content immunofluorescence setting; the subsequent screen will aim to identify compound ‘hits’ that restore or ameliorate this patient-specific phenotype. One main limiting factor in iPSC technology is line variability but, especially in monogenic disorders, the use of multiple patient lines and appropriate age-matched controls (including isogenic controls generated via CRISPR genome editing) allow for robust phenotypic assay set-up and results in downstream experiments [13]. In the protocol described, an autophagy (LC3)-based assay was used for the iPSC-derived neuronal screening; however, the workflow can be adapted to analysis of other proteins of interests. For the drug screening, access to an automated liquid handling system, an acoustic dispenser and a high-content imaging platform is required. Finally, for image analysis, bioinformatics support is recommended.

## 2 Materials

### 2.1 iPSCs

Characterize all iPSC lines to ensure true pluripotency prior to neuronal differentiation and screening experiments. Pluripotency characterization includes:

a) expression of pluripotency markers via immunofluorescence and RT-PCR,
b) Epi-Pluri-Score [20] (examining DNA-methylation levels at specific CpG sites),
c) spontaneous *in vitro* differentiation into all three germ layers,
d) maintenance of genomic integrity after reprogramming (e.g. SNP array analysis to ensure no large pathogenic deletions/ duplications have been acquired, and genomic DNA sequencing of targeted areas to ensure disease-causing mutations are maintained) and
e) non-integrating vector (e.g. Sendai virus) clearance, if relevant reprogramming methods have been used [17,18] (Notes 2–5).

### 2.2 Reagents, media, plasticware

Example manufacturers and catalogue numbers are provided below (Note 1).

#### 2.2.1 For iPSC maintenance

1 6-well Tissue Culture-treated plates [Sigma (CLS3516–50EA)]
2 mTeSR1, 5x mTeSR1 supplement, [StemCell technologies (85,870)] OR
3 TeSR-E8 Basal Medium and TeSR-E8 25X Supplement [StemCell Technologies (05,990)] (Note 6)
4 Penicillin-Streptomycin (stock concentration 10,000 U/ml) [ThermoFisher (15,140,122)]
5 Matrigel hESC-Qualified Matrix, LDEV-free [Corning (354,277)] (Note 6) OR
6 Vitronectin XF [StemCell Technologies (07,180)] (Note 6)
7 Ethylenediaminetetraacetic acid (EDTA) 0.02% solution [Sigma (E8008)]
8 Knockout Dulbecco's Modified Eagle's Medium (DMEM) [ThermoFisher (10,829,018)]
9 1x Dulbecco's Phosphate Buffered Saline (DPBS) [ThermoFisher (14,190,250)]
10 Gelatine from Bovine skin [Sigma (G9391)] 0.1% in Milli-Q water
11 TrypLE [ThermoFisher (12,563,029)]
12 p160-Rho-associated coiled-coil kinase (ROCK)-Inhibitor Thiazovivin [StemCell Technologies (72,252)]; stock concentration 10 mM in Dimethyl Sulfoxide [DMSO; Sigma (D8418)]
13 ReLeSR [StemCell Technologies (05,873)]
14 MycoAlert™ Mycoplasma Detection Kit [Lonza (LT07–218)]

#### 2.2.2 For neuronal differentiation

1 10-cm sterile non-adherent Petri dishes [e.g. Merck (CLS3262)]
2 12-well Tissue Culture-treated plates [Sigma (CLS3513–50EA)]
3 96-well low skirted SBS footprint microplates. E.g. CellCarrier-96 Ultra [PerkinElmer (6,055,300)]
4 Poly-L-ornithine hydrobromide (PO) [Sigma (P3655)]
5 Fibronectin (Fn) [ThermoFisher (33,010,018)]
6 Laminin (LN) [Sigma (L2020–1MG)]
7 1x DPBS
8 TrypLE
9 Accumax [Sigma (A7089)]
10 Knockout DMEM
11 Knockout-Serum Replacement [ThermoFisher (10,828,018)]
12 L-glutamine [ThermoFisher (2,503,081)]
13 Non-Essential Amino Acids 100x [ThermoFisher (11,140,035)]
14 2-Mercaptoethanol [ThermoFisher (31,350,010)]
15 Bovine Serum Albumin (BSA) [Tocris (5217)]

**Fig. 1:**
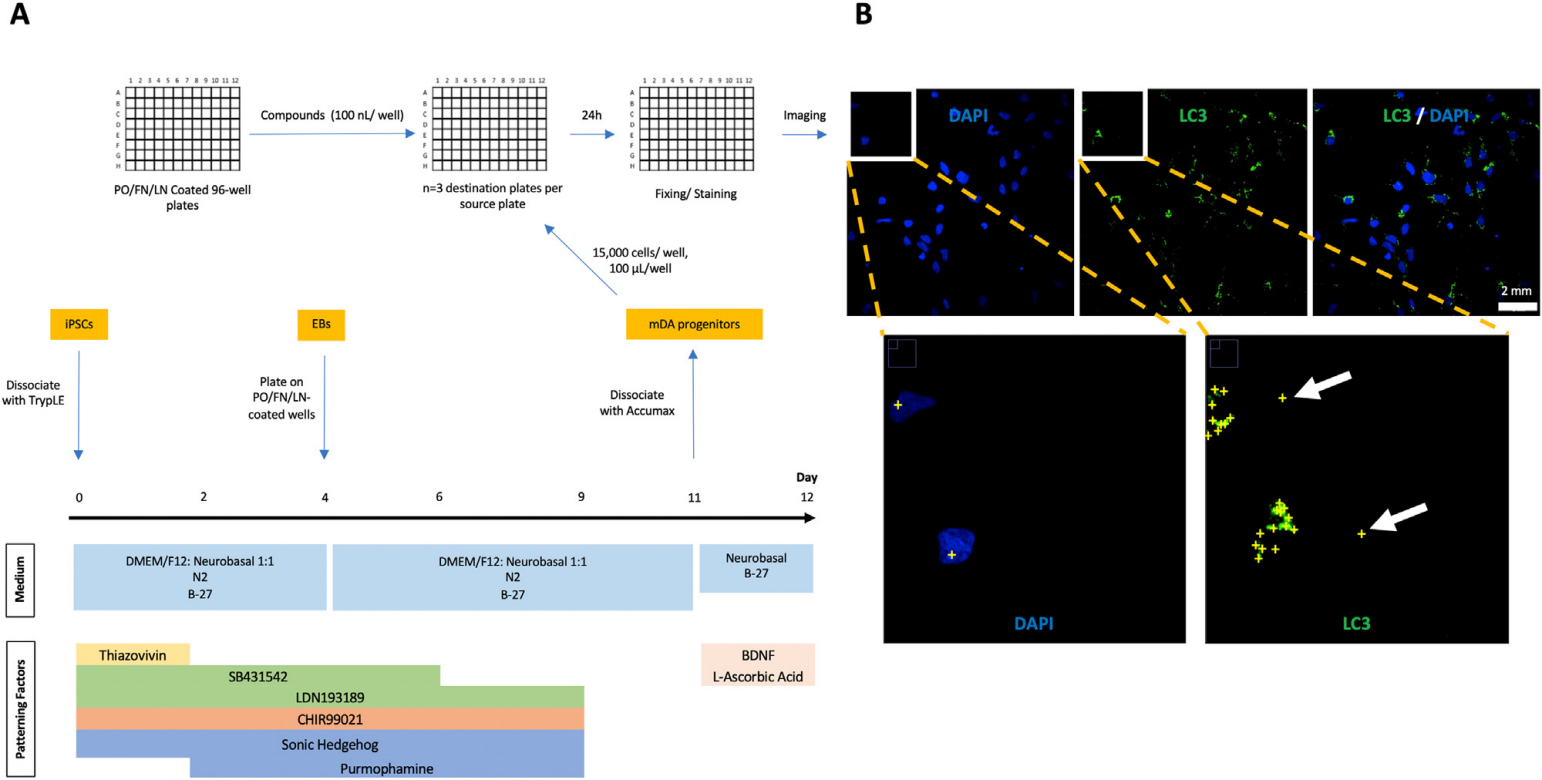
**Overview of mDA progenitor screening protocol. A. mDA neuronal differentiation.** Basal media and patterning factors required for each stage of the process are shown. On Day 11, compounds are dispensed from the source plates onto PO/FN/LN-coated, dried, plates. Subsequently, MDa progenitors are plated and cultured for another 24 h prior to fixing and staining. **B. Image Analysis.** Representative images. LC3 puncta and nuclei are counted for each imaged field, and LC3 puncta / nuclei values are calculated. Through image segmentation, LC3 puncta outside the cytoplasm (background noise) are excluded from the analysis. Abbreviations: EBs: embryoid bodies, FN: fibronectin, LN: laminin, mDA: midbrain dopaminergic, PO: poly-L-ornithin.

Additionally, the media and factors needed for each stage of differentiation are detailed in Table 1, Fig. 1A, and the Procedure section.

**Table 1:** *Neuronal differentiation media constitution*. Shaded rows indicate constituents removed or added on specific days during the mDA neuronal differentiation protocol.

**mDA Neuronal Differentiation Protocol**		
**Medium**	**Constituents (and stock concentrations, where applicable)**	**Dilution/ Final Concentration**
**Embryoid Body medium(D0-D4)**	**DMEM/F12**, with L-Glutamine, without HEPES [ThermoFisher (11,320–074)]: **Neurobasal** medium [ThermoFisher (21,103,049)]	1:1
	**N2 supplements** [ThermoFisher (17,502,048)]	1:100
	**B-27 supplements** [ThermoFisher (12,587–010)]	1:50
	**L-Glutamine**	2 mM
	**Penicillin/ Streptomycin** 10,000 U/ml	100 U/ ml
	**Thiazovivin** (on Days 0–2, only); stock 10 mM in DMSO	10 μM
	**SB431542** [Cell Guidance Systems (sm33–10)]; stock 10 mM in DMSO	10 μM
	**LDN193189** [Sigma (SML0559–5MG)]; stock 10 mM in DMSO	100 nM
	**CHIR99021** [Tocris Bioscience (4423)]; stock 10 mM in DMSO	0,8 μM
	**Sonic Hedgehog** C24II, Recombinant modified human [R&D Systems (1845-SH-025)]; stock 25 μg/ml in DPBS, 0.1% BSA	100 ng/ml
	**Purmophamine** [Cayman Chemicals (10,009,634–1 mg-CAY)] (Day 2-Day 9); stock 10 mM in DMSO	0.5 μM
**Neuronal Differentiation medium(D4-D11)**	**DMEM/F12:Neurobasal** medium	1:1
	**N2 supplements**	1:200
	**B-27 supplements**	1:100
	**L-Glutamine**	2 mM
	**Penicillin/ Streptomycin** 10,000 U/ml	100 U/ ml
	**SB431542** (out on D6)	10 μM
	**LDN193189** (out on D9)	100 nM
	**CHIR99021** (out on D9)	0,8 μM
	**SHH** (out on D9)	100 ng/ml
	**Purmophamine** (out on D9)	0.5 μM
**Final Differentiation medium(D11 onward)**	**Neurobasal medium**	1
	**B-27 supplements**	1:50
	**L-Glutamine**	2 mM
	**Penicillin/ Streptomycin** 10,000 U/ml	1:100
	**L-Ascorbic Acid** [Sigma (A4403–100MG)]; stock 50 mM in H20	0,2 mM
	**BDNF** [Miltenyi (130–093–811)]; stock 5 μg/ml in DPBS, 0.1% BSA	20 ng/ml

#### 2.2.3 Immunostaining and drug screening

1 Small molecule library source plates, such as Beckman Echo LDV plates
2 Bafilomycin A1 [Sigma (B1793)]
3 DMSO
4 4% Paraformaldehyde (PFA) [Santa Cruz (30,525–89–4)]
5 1x DPBS
6 NH_4_Cl [Sigma (A9434–500 G)], 50 mM in DPBS
7 Ice-cold methanol (−20 °C) [e.g., Sigma (34,860–1L-R)]
8 Foetal Bovine Serum (FBS) [Sigma (F9665)]
9 Triton 100X [Sigma (T8787–100ML)]
10 Primary and Secondary antibodies:
  a LC3B rabbit monoclonal antibody (clone D11), Cell Signalling Technology (3868S), 1:200 dilution.
  b Alexa Fluor 488 Goat Anti-Mouse IgG, ThermoFisher (A-11,001), 1:400 dilution].
11 DAPI (4′,6-diamidino-2-phenylindole) [Sigma (28,718–90–3)]

#### 2.2.4 Equipment

1 Class 2 biological safety cabinets
2 Tissue culture CO2 incubators with humidified atmosphere and supply of 5% CO_2_ at 37 °C
3 Labcyte Echo550 Liquid Handler, or similar
4 Agilent Biotek Multi-Flo Multi-Mode Dispenser, or similar
5 Beckman Coulter Biomek NX^P^ Automated Workstation, or similar
6 Agilent BioTek EL406 Washer Dispenser, or similar
7 PerkinElmer Opera Phenix confocal microscope, or similar
8 PerkinElmer Plate Handler II, or similar

As an alternative to the Opera Phenix microscope, other high content imaging platforms can be used, e.g. the Thermo Scientific CellInsight CX7 Pro High-Content Screening (HCS) Platform. As an alternative to the Echo520 or Echo550 accoustic dispensers, standard liquid handling robots could be used. This may require additional dilution steps to prepare the right stock concentrations. Similarly, other high-content imaging platforms can be used and may require adjustments in the protocol setup (Section 2.3.).

#### 2.2.5 Software

1 Beckman Coulter SAMI EX scheduling software, or similar
2 PerkinElmer Harmony High-Content Imaging and Analysis Software, or similar
3 PerkinElmer Columbus Image Data Storage and Analysis System, or similar
4 ImageJ
5 R
6 Microsoft Excel

As an alternative to IMAGEJ, other image analysis software can be used such as CellProfiler [21], Icy or commercial software that is often integrated into the hardware of the high-content imaging platform (Section 2.5.).

## 3 Procedure

Carry out all experiments at room temperature unless otherwise specified.

### 3.1 iPSC maintenance

1 To prepare iPSC maintenance media, add 5x mTeSR1 supplements (100 ml) to 400 ml of mTeSR1 basal medium, as per manufacturer's instructions. Supplement medium with 1% Penicillin-Streptomycin (stock concentration 10,000 U/ml; final concentration 100 U/ml). Alternatively, for TeSR-E8, mix TeSR-E8 Basal Medium (480 mL) with TeSR-E8 25X Supplement (20 ml) and 1% Penicillin-Streptomycin (100 U/ml).
2 To coat 6-well plates with Matrigel, resuspend 10 mg/ml of Matrigel in 25 ml Knockout DMEM (Note 6). Plate 1 ml in wells of a 6-well plate and incubate for at least 1 hour at 37 °C. For Vitronectin XF coating, dilute 1 ml of Vitronectin XF in 25 ml of sterile 1 x DPBS, then plate onto 1 ml/ well on 6-well plates for at least 1 hour at 37 °C. Aspirate and discard coating solution just before plating the passaged iPSCs. Avoid trying of coted plates.
3 When thawing iPSCs, thaw cryovial contents at 37 °C. When partly thawed, resuspend in 10 ml minimum of culture medium (mTeSR1 or TeSR-E8), in a 15 ml Falcon tube and centrifuge at 300 g for 5 min. Resuspend in culture medium supplemented with thiazovivin (stock concentration 10 mM in DMSO, 10 μM final concentration) in ratio of one cryovial per one well of a 6 well plate, and plate on Matrigel- or Vitronectin XF-coated 6-well plate. After 24 h, change to iPSCs maintenance medium without thiazovivin (Note 9).
4 Maintain iPSCs in mTeSR1 on 6-well, Tissue Culture-treated, Matrigel-coated plates. Alternatively, use TeSR-E8 on Vitronectin XF-coated plates. Change media daily [22,23] (2 ml per 6-well plate well) and regularly passage (when confluent; approximately every 4–7 days depending on each iPSC line and cell seeding ratios) (Note 10).
5 To passage iPSC lines, wash cells in 1x DPBS (2 ml/ well of a 6-well plate) and add 1 ml EDTA 0.02%. After approximately 5 min of incubation at 37 °C, aspirate EDTA. iPSCs should be gently lifted in clumps of 5 to 10 cells using sequential washing of separate sections of the well from low to high, with 1 ml culture media each for each wash, and using a P1000 pipette. Avoid contact of cells with media if not ready to be dissociated, as this would re-accumulate Ca^2+^ and thus cell-cell adhesion. Then, transfer to a sterile Falcon tube and, finally, to new Matrigel (or Vitronectin XF)-coated plates. The ratio of passaging is usually 1 well into 2 wells for cells cultured in the mTESR1/Matrigel system. For TeSR-E8/ Vitronectin XF maintenance, consider ratios of 1 well into 3–4 wells, especially if spontaneous differentiation is prominent (Note 11).
6 To remove spontaneously differentiating cells from iPSC cultures, choose from the options below (Note 12):

6A. For iPSCs cultures on Vitronectin-coated plates: after EDTA application, do not dissociate iPSCs with a P1000 pipette. Instead, slowly add 1 ml of medium to each well, and gently tap the sides of the plate to dissociate clumps of undifferentiated iPSCs and allow differentiating cells to remain attached. Leave newly dissociated iPSCs to sediment to the bottom of a 15 ml Falcon tube filled with culture medium for 3–4 min; subsequently, aspirate and discard most of the supernatant (containing differentiating single cells) and only plate the bottom 2 ml (containing undifferentiated iPSC clumps), at a final volume of 2 ml/ well.

6B Use ReLeSR (main method for iPSCs cultured on the matrigel/ mTeSR system). Wash iPSCs with 1x DBPS and apply ReLeSR (1 ml per well of a 6-well plate) for 1 min. Then, aspirate ReLeSR and leave wells to dry out for 4 min at room temperature (optimal time will depend on the cell type; cells are ready when clear sign of cell-cell detaching are visible). Subsequently, dissociate non-differentiating iPSC colonies by adding 1 ml/well of culture medium and gently tapping the plate, then plate onto newly coated plates (see manufacturer protocol).

6C. Use the gelatine 0.1% method. Coat 6-well plates with sterile 0.1% gelatine in Milli-Q water and incubate at 37 °C for 1–24 h. Harvest iPSC lines exhibiting obvious spontaneous differentiation using TrypLE, centrifuge at 300 g for 5 min, re-suspend the pellet in mTeSR1 and Thiazovivin (stock concentration 10 mM in DMSO, diluted 1:1000 in culture medium to a final concentration of 10 µM), seed on the gelatine-coated wells and incubate at 37 °C for 30 min (allowing for differentiating cells to adhere to the bottom of the plate). Subsequently, aspirate the medium (containing un-differentiated iPSCs) and plate onto Matrigel-coated plates. Time of seeding onto gelatine-coated dishes might be extended to maximum 40 min.

1 To freeze and store iPSCs, dissociate cells when confluent with TrypLE, centrifuge at 300 g for 5 min and resuspend contents of one well of a 6-well plate in 1 ml of 90% KOSR complete medium and 10% DMSO per cryovial. Store vials in a freezing container, e.g., Nalgene® Mr. Frosty (Sigma Aldrich C1562–1EA) or similar, at −80 °C for 24 h before transferring to liquid nitrogen. Note: frozen iPSCs can be stored at −80 °C for a maximum of three months; longer time will severely affect cell survival after thawing.

### 3.2 Differentiation into dopaminergic progenitor cells

1 On Day 0, to form Embryoid Bodies (EBs), harvest iPSCs: aspirate medium, wash with 1x DPBS (2 ml/ well), apply TrypLE (1 ml/ well) and incubate at 37 °C for 5 min. Then, aspirate TrypLE, dissociate with mTeSR1/ TeSR-E8 using a P1000 pipette, transfer to a 15 ml Falcon tube, and centrifuge at 300 g for 5 min. Count the number of total cells harvested, resuspend 8 × 10^6^ cells in 11 ml of differentiation medium and plate onto one non-adherent 10 cm Petri dish (ratio: 8E6 cells/10 cm petri dish). Note: if using a smaller petri dish, scale down the number of cells to be plated (e.g. for a 6 cm Petri dish, seed 4 × 10^6^ cells).
2 Change media every 48 h for the duration of the differentiation. From Day 0 to Day 3, culture cells in DMEM/F12:Neurobasal (1:1), N2 supplement (1:100), B27 minus vitamin A (1:50), 2 mM L-glutamine, and Penicillin-Streptomycin (1:100; stock concentration 10,000 U/ml).

From Day 4 to Day 10, culture cells in DMEM/F12:Neurobasal (1:1), N2 (1:200), B27 minus vitamin A (1:100), 2 mM L-glutamine and Penicillin-Streptomycin (1:100). Moreover, supplement culture medium as follows (Table 1, Fig. 1A, Note 14):

2A. For the first two days (Days 0 to 1), use thiazovivin 10 µM, to aid the survival and self-renewal of dissociated iPSCs  [24,25].

2B To induce neural identities via dual SMAD inhibition, supplement medium with 100 nM LDN193189 (Bone Morphogenic Pathway inhibitor) for 9 days (day 0 to day 8), and with 10 μM SB431542 (TGF beta blocker) for 6 days (day 0 to day 5) [26]. For LDN193189, to minimise pipetting errors, pre-dilute stock solution by 1:100 in neurobasal medium, and then further dilute this by 1:1000 into the final differentiation medium.

2C. Use Sonic hedgehog (SHH-C24II) 100 ng/ml for 9 days (Day 0 to day 8) and the SHH pathway activator Purmorphamine 0.5 μM for 7 days (Day 2 to Day 8) to guide the differentiating cells towards floorplate identities [27]. For purmorphamine, to minimise pipetting errors, pre-dilute stock solution by 1:100 in neurobasal medium, and then further dilute this by 1:200 into the final differentiation medium.

2D. Use CHIR 99,021 0.8 µM (GSK3 inhibitor) for 9 days (Day 0 to day 8), to activate the WNT1 pathway and guide cells towards midbrain fate.

1 For Days 11 and 12, culture cells in medium including Neurobasal B27 minus vitamin A (1:50), 2 mM L-glutamine, 0.2 mM ascorbic acid (AA), and 20 ng/ml BDNF to help the development and survival of mDA progenitors [28].
2 For Day 4 EB plating, perform coating of 12-well plates with PO/Fn/LN as follows:

On Day 0, add PO 15 µg/ml, in 1x DPBS, to well and incubate at 37 °C for 48 h (700 µl per well of a 12-well plate, or 70 μl/well of a 96-well plate). After 48 h (Day 2), wash with 1x DPBS and add Fn/LN, each diluted at 5 µg/ml in 1x DPBS. Incubate plates at 37 °C for 48 h, then aspirate and dry wells prior to cell seeding.

**Fig. 2:**
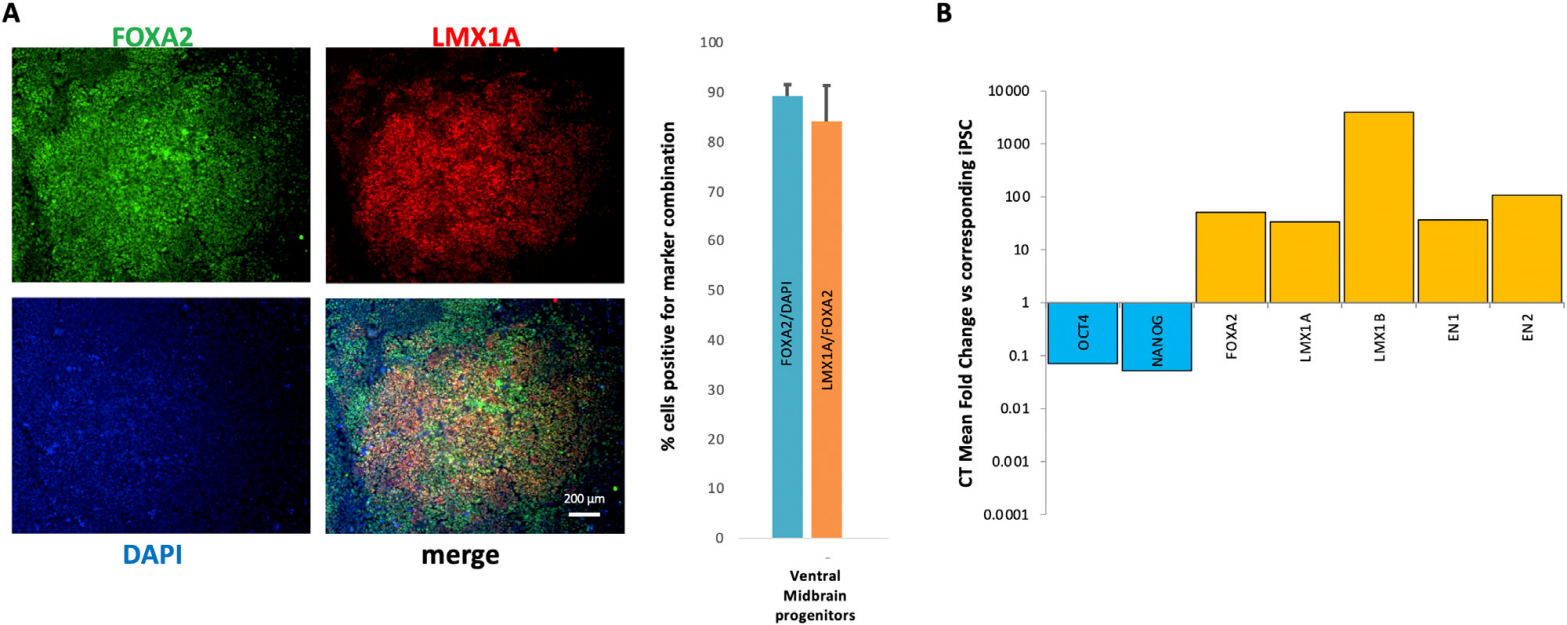
**Characterization of ventral midbrain progenitors at Day 11 of differentiation. A. Immunofluorescence.** Expression of ventral midbrain markers forkhead box protein A2 (FOXA2) and homeobox transcription factor 1 alpha (LMX1A) at Day 11 of differentiation. Co-expression of FOXA2 and LMX1A is thought to be unique to the ventral midbrain, hence a high percentage of doubly-positive cells indicates effective initiation of differentiation into mDA neurons. Quantification via counting doubly-positive cells (e.g., on ImageJ) is recommended; the relevant quantification graph suggests effective differentiation into ventral midbrain progenitors as per relevant literature [17,18]. **B. Real-Time Quantitative Reverse Transcription PCR (qRT PCR).** Day 11ventral midbrain progenitors tested for appropriate gene expression suggestive of effective initiation of mDA neuronal differentiation. For each marker/ target, $\Delta C_{T}\;$mean values on Day 11 (calculated using the target's $C_{T}$ mean values, normalised versus the ones from the housekeeping gene GAPDH) are compared to ones in corresponding iPSCs. The line exhibits downregulation in gene expression of pluripotency-related markers *OCT4* and *NANOG*, as well as upregulation in gene expression of ventral midbrain markers *FOXA2, LMX1A, LMX1B, EN1* and *EN2* . When such immunofluorescence marker ratios and qRT PCR marker patterns are achieved, one can be confident of effective generation of ventral midbrain progenitors; if the differentiation shows different patterns, cells must be discarded, and new differentiations started.

1 On day 4, plate EBs onto 12-well plates pre-coated with PO/Fn/LN. Transfer cells and medium from the 10-cm dish into a 15 ml Falcon tube, centrifuge at 200 g for 1 min, then resuspend in 6 ml of Day 4 medium and plate (1 ml/ well of a 12-well plate).
2 On Day 11 of differentiation, robust characterisation (via immunofluorescence and Real-Time Quantitative Reverse Transcription PCR) is needed prior to downstream experiments, to ensure generation of progenitors with true ventral midbrain identities [[17], [18], [29]] (Fig. 2).

### 3.3 Cell seeding and compound administration (Fig. 1A, Fig. 3)

1 Coat the 96-well low skirted microplates with PO/Fn/LN using the Beckman Coulter Biomek NXp and Biotek EL406 automated workstation and the SAMI scheduling software, as follows:

On Day 7, add PO 15 µg/ml, in 1x DPBS, to well and incubate at 37 °C for 48 h (70 μl/well of a 96-well plate). After 48 h, wash with 1x DPBS (100 μl/ well) and add Fn/LN (70 μl/well), each diluted at 5 µg/ml in 1x DPBS. Incubate plates at 37 °C for 48 h, then aspirate and dry wells prior to cell seeding.

**Fig. 3:**
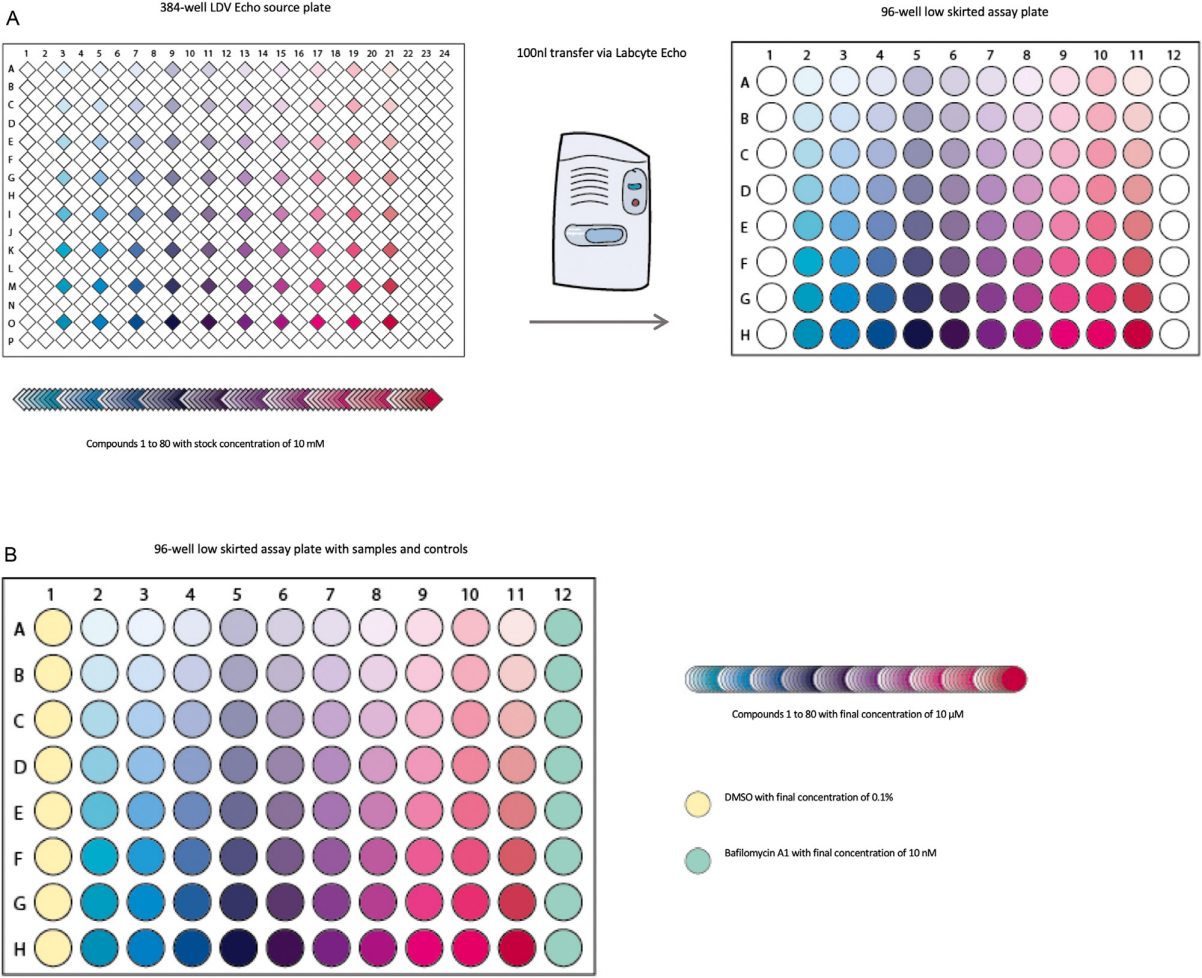
**Automated compound delivery. A.** Schematic drawing of the 384 LDV Echo source plate containing the first 80 individual drugs of a compound library. The library can be dispensed into 96 well assay plates using the Labcyte Echo 550 Acoustic dispenser. **B.** Final plate layout in the 96 well assay plate containing controls and library compounds.

1 On Day 11 of differentiation, dry PO/Fn/LN-coated 96-well low skirted destination microplates.
2 Administer compounds of the library of choice to the coated assay plates using the Labcyte Echo 550 Liquid Handler:

Library compounds with a stock concentration of 10 mM are located in every second well of a 384 LDV Echo plate. To achieve a final concentration of 10 µM in the 96 well assay plates, transfer 100 nl of the compounds from the 384 LDV plates into the coated 96 well plates using the Labcyte Echo Dose-Response software (Fig. 3A). Here one can compress the intermitted pattern of the 384 well source plate by defining every second well in columns 3 to 21 of the source plate and every well in columns 2 to 11 in the destination plate as samples. For a one-to-one transfer provide a single source concentration and transfer volume and select one data point per curve. Then, dispense 100 nl of negative and positive controls. For LC3-based high content screening, use DMSO as negative control in Column 1 and Bafilomycin A1 (stock concentration 10 µM, final concentration 10 nM after adding the cell suspension) as positive control in Column 12 (Fig. 3B). If stock solutions of both controls are positioned in the library source plate itself, select the respective wells in the source plate and define column 1 and 12 in the destination plate as controls within the Echo Dose-Response protocol.

1 To test each compound in triplicates, dispense the compounds and controls of one 384 well source plate into three 96-well destination plates. Further automation using integrated stackers, a microplate delidder, and a robotic arm controlled by the Tempo software facilitate the drug dispensing process.
2 To dissociate Day 11 cells from 12-well plates, firstly make KOSR complete medium. Medium composition (500 ml): 390 ml Knockout DMEM, 100 ml Knockout-Serum Replacement (20%), 5 ml l-glutamine (2 mM), 5 ml Non-Essential Amino Acids 100x (1%), 500 µl 2-Mercaptoethanol (50 mM).
3 Wash Day 11 cells with 1x DPBS and incubate with 500 μl of Accumax for 20 min at 37 °C. Add KOSR complete medium to stop the enzymatic reaction, dissociate cells with a P1000 pipette and pellet by transferring in a KOSR-containing 15 ml Falcon tube and centrifuging at 300 g for 5 min. *Re*-suspended in Day 11 medium at a dilution of 15,000 cells/ml. Seed 100 μl per well (15,000 cells/ well) in the 96-well low skirted destination microplates using the Biotek Multi-Flo Multi-Mode Dispenser.
4 Culture cells at 37 °C for 24 h, then fix and stain as described below (Section 3.4.).

### 3.4 Automated immunostaining

For automated immunostaining, use a washer and dispenser such as the Biotek EL406. This can be integrated into a larger liquid handler set up, with a Cytomat hotel and the Beckman Biomek NXp liquid handler including an Inheco cooling unit. Set up scripts for the individual dispensing and washing steps and run using the SAMI Ex scheduling software. Move plates to the individual stations in the protocol using a Scara robotic arm (Tables 2 and 3).

**Table 2:** **Automated PFA fixation.** The stations the assay plates run through during the fixation process are listed in the left column. The individual steps of the fixation protocol at the respective stations are described in the right column.

Station	Step
Cytomat Hotel	Home position
Plate Washer	Aspirate growing media with washer head at travel rate 1
	Dispense 80 µl of 4% PFA with syringe A at flow rate 1
Cytomat Hotel	Incubate plate at room temperature and move plate back to plate washer within 17 min
Plate Washer	Wash with 1 x DPBS using the washer head, in 3 cycles:Aspirate at travel rate 1Dispense 100 µl of 1x DPBS at flow rate 1
Cytomat Hotel	Home position. Plates will be placed here, ready for subsequent steps of the staining protocol or for collection and intermediate storage

**Table 3:** **Automated cell staining.** Stations within the imaging set up are listed on the left. The respective steps for cell permeabilization, blocking, washes and primary antibody **(A)** as well as secondary antibody administration **(B)** are listed on the right.

A	DAY 1
Place	Step
Cytomat hotel	Home position
Plate washer	Aspirate PBS with washer head at travel rate 1
	Dispense 80 µl of ice-cold methanol with syringe A at flow rate 1
Inheco Cooling Unit	Incubate plate at 4 °C and move plate back to plate washer within 17 min
Plate Washer	Wash with PBS using the washer head in 3 cycles:Aspirate at travel rate 1Dispense 100 µl of PBS at flow rate 1
Plate Washer	Aspirate PBS with washer head at travel rate 1Dispense 50 µl of blocking buffer with syringe B at flow rate 1
Cytomat Hotel	Incubate plate at room temperature and move plate back to plate washer within 65 min
Plate Washer	Aspirate blocking buffer with washer head at travel rate 1 CWDispense 40 µl of primary antibody solution with Peri-pump dispenser at low flow rate
Cytomat Hotel	Home position. Plates will be placed here, ready for collection to be stored at 4 °C overnight
B	**DAY 2**
Place	Step
Cytomat Hotel	Home position
	Wash with PBS using the washer head in 3 cycles:Aspirate at travel rate 1Dispense 100 µl of PBS at flow rate 1
Plate Washer	Wash with PBS using the washer head in 3 cycles:Aspirate at travel rate 1Dispense 100 µl of PBS at flow rate 1
Plate Washer	Aspirate DPBS with washer head at travel rate 1 CWDispense 40 µl of secondary antibody solution with Peri-pump dispenser at low flow rate
Cytomat Hotel	Incubate plate at room temperature and move plate back to plate washer within 65 min
Plate Washer	Wash with PBS using the washer head in 3 cycles:Aspirate at travel rate 1Dispense 100 µl of PBS at flow rate 1
Cytomat Hotel	Home position. Plates will be placed here, ready for imaging

Set up the following protocol using the liquid handler pipeline listed below the fixation/staining steps (Tables 2 and 3):

1. Fix with 4% PFA, as described in Table 2.

2. Permeabilize and fix a second time in ice-cold methanol (kept at −20 °C) for 15 min at +4 °C, then wash three times with 1 x DPBS.

3. Incubate in blocking buffer (1 x DBPS, 10% FBS and 0.1% Triton) for 1 h at room temperature.

4. Incubate destination plates overnight at 4 °C with primary anti-LC3 antibody (1:200 dilution) diluted in blocking buffer (50 μl/well).

5. Incubate cells with secondary antibody (1:400 dilution) in blocking buffer (50 μl/well) and DAPI (1:1000) for 1 h at room temperature. Wash three times with 1 x DPBS, then store at +4 °C, in the dark, until image acquisition.

### 3.5 Image acquisition

1. For imaging, use the PerkinElmer Opera Phenix confocal microscope (**Note 7**).

2. For image acquisition, use the Harmony software provided with the PerkinElmer Opera Phenix. For the set-up, follow steps below:

2A. Choose the appropriate plate type from the drop-down menu. Ideally choose a low skirted 96 well plate for the objective to be able to move into the far corners of the plate.

2B Choose a two peak autofocus.

2C. To obtain high resolution images, use a water objective with high numerical aperture in confocal mode and a binning setting of 1. We obtained good results using the 40x water objective (Aperture 1.1).

2D. Select the appropriate channels for your staining. In the Harmony software, the pre-set Hoechst channel and the Alexa 488 channel can be used.

Hoechst 33,342: 375 nm excitation laser, 435–480 nm emission filter

Alexa 488: 488 nm excitation laser and 500–550 nm emission filter

2E. Adopt the pre-set exposure time, laser power and height settings to check individual wells for cells using the “snapshot” function.

2F. One can identify the most suitable plane within the neuronal progenitor cultures by setting up a stack in the “Layout Selection” tab: For one-layered cultures on low skirted plates with a bottom thickness of around 0.18 mm, one can start with a bottom plane of −5 µm and go up in increments of 0.5 µm to reach a top plane of 5 µm. By switching from the “Define Layout” tab to the “Image” tab, one can now browse through the stack and define the section with highly resolved LC3 spots. Manually transfer the height of the preferred section into your channel setting.

2 G. To determine the most appropriate exposure settings, pick a well containing a positive control in the “Define Layout” tab. Increase power and exposure time to ideally reach a pixel intensity of around 9000 for LC3 spots and nuclei. Pixel intensity of individual spots can be checked during the set up process (right click, choose: show intensity, click on the desired spot). We previously acquired images with the following settings:

Hoechst 33,342: time = 300 ms, power = 100%, height = −1.0

Alexa 488: time = 1000 ms, power = 100%, height = −1.0

2H. To avoid ‘bleed-though’ between channels, separate both channels with the “Channel Sequence” option.

2I. Select all wells, and 20 fields per well, to be imaged.

3. For further automation a rack for plate storage and a robotic arm for plate feeding into the microscope can be used. When using the PerkinElmer Opera Phenix these will be under the control of the plate::works™ software. Within the plate::works™ software, set up your “method” (the path the individual plate takes through the imaging set up). For fixed and stained assay plates of the LC3 screen, this will be: Rack => Barcode Reader => Phenix => Rack. Define the plate count, experiment name and owner as specified earlier in the Harmony software, as well as duration of imaging. Fill the stack with the defined amount of assay plates. Within the “Run Experiment” window in the Harmony software, switch to the “Remote” control and start the imaging process.

4. Upon completion, export captured data to the Columbus Image Data Storage and Analysis System and, subsequently, as .TIF images to institutional secure drives as required.

### 3.6 Image analysis (Fig. 1B)

Process images using a high-content analysis computer equipped with at least 32 GB memory, ideally a Supermicro SuperServer 4048B-TR4FT system with 1 TB memory that we used and a recent ImageJ version (we used version 1.52) (Rasband, W.S., ImageJ, U. S. National Institutes of Health, Bethesda, Maryland, USA, https://imagej.nih.gov/ij/, 1997–2022.). Analyse a full plate of 1920 images (96 wells x 20 field of views) (25 GB) as a set on each ImageJ instance using a custom ImageJ macro with a modified workflow from already published work [30]:

1 Read the image set of a plate into the memory and convert it into a hyperstack. Perform iterations of the calculation as the following:
2 Apply a Mean filter with 20 pixels radius parameter on the nuclear channel (Channel 1: 375 nm) for image pre-processing.
3 Segment nuclei using a watershed segmentation algorithm with appropriately optimized parameter that quantifies the nuclei number based on the local maximum pixel intensities.
4 Apply a watershed segmentation to the LC3 channel (Channel 2: 488 nm) without any pre-processing steps to quantify the number of LC3 puncta.
5 Calculate the LC3 puncta / nuclei ratio per well using an R scri pt R[31] (https://www.r-project.org/, we used R version 3.5.2).
6 Process the resulting data using the Bioconductor R package cellHTS2[32] version 2.46.1. Normalize data using the negative control values and transform to Z scores.
7 Calculate the mean value of the 3 replicates for the final, single Z score per treatment (**Note 15**).

### 3.7 Flow diagram

A schematic summarizing the iPSC neuronal differentiation, seeding of ventral midbrain progenitors onto multiwell plates for compound screening, and image analysis is provided in Fig. 1.

### 3.8 Troubleshooting/ notes

**Fig. 4:**
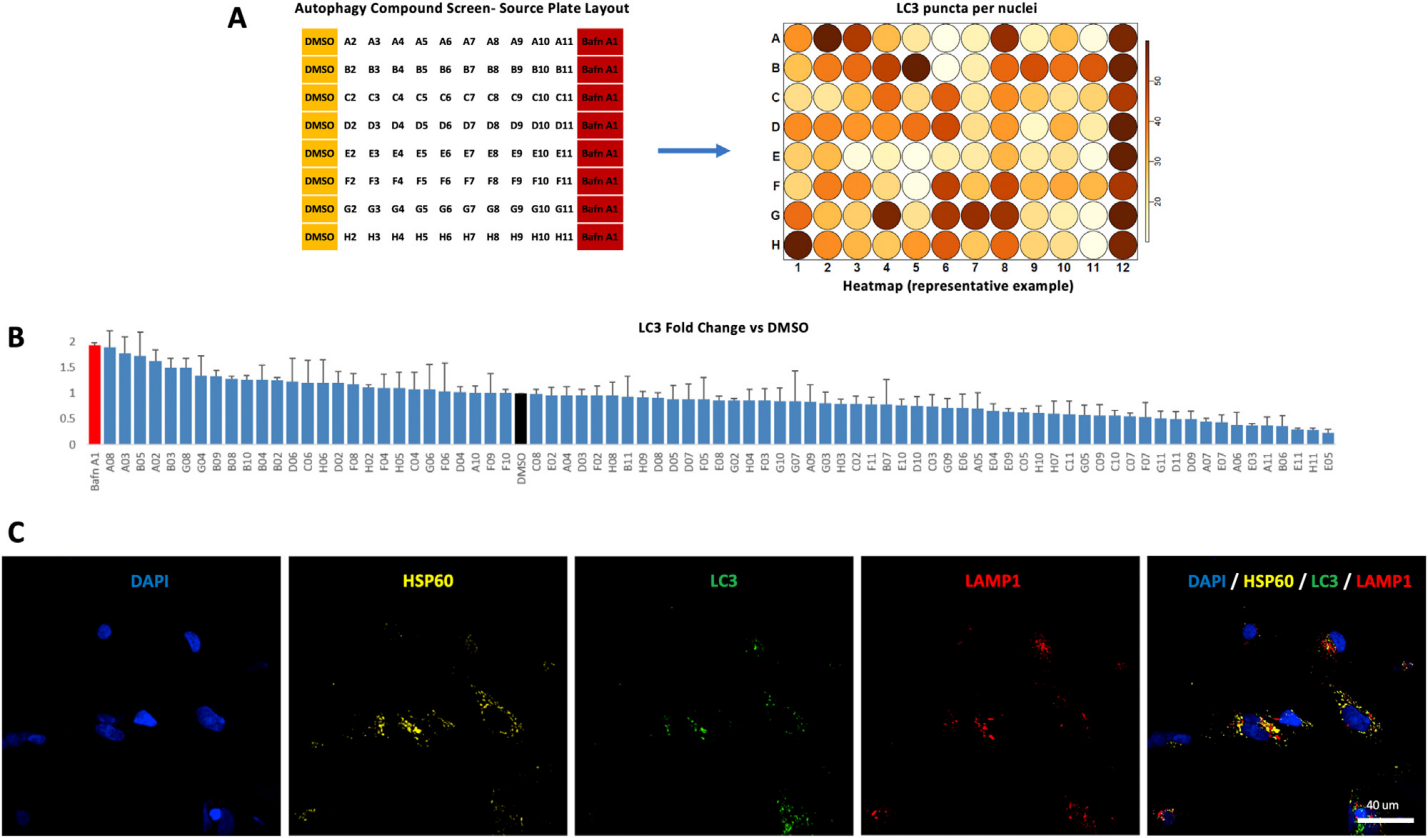
*Assay reproducibility and capacity for high content. A***.** To assess reproducibility, a pilot source plate was created containing i) compounds known to enhance LC3 puncta production (either by inducing autophagy flux or blocking autophagosome to lysosome fusion), ii) compounds with no expected effect on LC3 puncta, as well as iii) negative and positive controls (DMSO and Bafilomycin A1 in columns 1 and 12, respectively). After treatment with these compounds, destination plates in technical triplicates were fixed, stained and imaged. Image analysis generated heatmaps depicting increasing LC3 puncta per nuclei numbers in darker shades of orange. **B.** Compound treatments reproducibly generated similar numbers of LC3 puncta per nuclei in all technical replicates. In detail, we observed standard deviation for the 3 replicates ranging from 0.028 and 0.592, with mean and median values of 0.209 and 0.157 respectively (attesting to the reproducibility of the assay). **C.** The assay can be used to image multiple stains simultaneously [e.g., nuclear stains (DAPI), mitochondrial markers (HSP60), autophagy (LC3) and lysosomal (LAMP1) markers] and their response to different treatments, hence providing multiple parameters that can be useful for secondary hit validation. Apart from puncta numbers, multiple other parameters can also be analysed, such as cell morphology, spot area, intensity and marker co-localization.
16 The screen is robust (see summary of triplicates of the pilot plate in Fig. 4A, B) and has been performed in multiple iPSC-derived neuronal progenitor lines, including iPSC-derived neuronal progenitor lines from patients with Beta-Propeller Associated Neurodegeneration (Papandreou et al., unpublished).
17 The described assay is high content-based. Hence, several other markers can be imaged simultaneously, and multiple outputs (e.g., puncta numbers, area, intensity, co-localization etc.) captured for each marker, which can in turn be used for secondary hit validation (Fig. 4C).

1 Prepare and store all media and reagents at the temperature specified by the manufacturer. Diligently follow waste disposal regulations when disposing materials.
2 For cell culture, work under sterile conditions in Class 2 biological safety cabinets at all times, to avoid sample contamination. Regularly test all cells for mycoplasma contamination, e.g. via the MycoAlert™ Mycoplasma Detection Kit, and immediately isolate or discard contaminated lines.
3 Discard all iPSC lines which show a spontaneous differentiation level above 5–10% and do not use for downstream differentiations (Section C1).
4 When setting up phenotypic assays for subsequent screening, use as many patient-derived and age-matched iPSC lines as possible; in cases of monogenic disorders, CRISPR/Cas9-generated isogenic controls (where the disease-causing mutation is substituted with the ‘wild-type’ sequence via Homology Directed Repair) should also be considered [17,33]. This approach helps overcome the inherent issue of line-to-line variability in iPSC models and ensures robust and reproducible results in downstream experiments (Section C1).
5 Maintain iPSC lines in culture for a maximum of 3 months after thawing (10–15 passages) to avoid acquisition of chromosomal/ genetic and epigenetic abnormalities that might influence results of downstream applications (Section C1).
6 Maintenance on mTeSR1/ matrigel or TeSR-E8/ Vitronectin largely depends on the conditions used during iPSC reprogramming. It is generally preferable to continue using the system the iPSCs were generated on; changes from one system to the other are also possible (but might exert stress on the cells and/ or result in unwanted spontaneous differentiation). If matrigel is unavailable or difficult to purchase, substitute with GeltreX, (e.g., A1569601 or A1413302 by ThermoFisher).
7 Thaw iPSCs as quickly as possible, at 37 °C, as slower thawing can result in reduced cell survival. Do not wait until the cryovial is fully thawed; instead, start resuspending in culture medium when a small frozen clump can still be seen (Section C1.3.).
8 Daily media change when maintaining iPSCs is crucial; it reduces cell stress, and the possibility of iPSCs starting to spontaneously differentiate, and promotes healthy and efficient downstream differentiations. Changing media daily prevents the pH (mainly dependent on confluence) from becoming too acidic, which in turn induces cellular stress. Moreover, maintenance media contain FGF2 which is crucial to maintain pluripotency but degrades very quickly at 37 °C. Stress and reduced FGF2 levels can trigger the release of differentiation factors into the medium (Section C1.4.).
9 Passage iPSCs when confluent. For cells cultured on Matrigel, there should be no gaps seen in the wells under microscopy. Conversely, in vitronectin-coated plates, iPSCs often grow in distinct, somewhat circular, colonies that do not expand to cover the whole well (some gaps between colonies are often present). For these, passage when the centre of the iPSC colonies becomes very dense (prolonging culture without passaging will stress the cells and lead to spontaneous differentiation) (Section C1.5.).
10 The method described in 6A. is the preferred, and usually sufficient, method for ‘cleaning’ iPSCs cultured on vitronectin-coated plates; here, undifferentiated cells form often-distinct colonies that can be detached by gentle tapping after incubation with EDTA. A (usually small) number of differentiating single cells can be seen around the iPSC colonies, which either do not detach with tapping or stay in the supernatant and can be removed by aspiration (with the clumps of iPSCs sedimenting at the bottom of the Falcon tube). Methods 6B. and 6C. can be used for iPSCs cultured on both Matrigel and vitronectin, and sometimes a combination of all methods (and repeated cleaning) might be required. In cases of persistent spontaneous differentiation, have low threshold of discarding the cells and thawing a new cryovial (Section C1.6.).
11 Number of starting cells for downstream mDA neuronal differentiation (approximately 8 million cells in each 10 cm dish) might be different for different iPSC lines. In case of low differentiation efficiency, proper cell number titration for each line should be considered (Section C2.1.). Moreover, differentiation efficiency might vary due to the concentration of specific patterning factors and, in the case of mDA neurons, particularly SHH and CHIR99021. Therefore, in case of low differentiation efficiency, also consider titration of these factors (Section C2.2.).
12 Many of these processes (e.g. SMAD inhibition, guidance to ventral midbrain fates), and relevant factor supplementations, occur in parallel. Please consult Table 1 and Fig. 1 for exact media constitutions for each stage of the neuronal differentiation process (Section C2.2.).
13 Where applicable, e.g. in cases of increased background signal/ non-cytoplasmic puncta numbers when performing image analysis, consider repeating the experiment (plate treatments, staining and image analysis) (Section 3.6.7.). Always check sample fields of view from random destination plates/ wells to ensure sufficient experiment quality.
14 Similar neuronal differentiation protocols (e.g. for cortical neurons [34,35]) can also be used. Ensure low density plating to allow accurate puncta per nuclei calculations, and/ or consider bioinformatics support when performing image analysis (Section C2).
15 In this method, we describe an autophagy-targeted screening assay based on LC3 immunofluorescence. However, other assays visualising a) a protein, protein complex, or protein modification, b) a cellular structure, e.g. an organelle, or a cellular process (e.g., cell death, protein trafficking etc.) can similarly be used as primary phenotypic assays for the drug screen (Fig. 4).

## 4 Anticipated results

The screen should identify small molecules with high Z scores, that restore or ameliorate the patient-specific phenotypic deficit (in this protocol, compounds that significantly enhance LC3 puncta production). These compound hits need to be further tested for any impact on cell viability. Their ability to restore other patient-specific cellular phenotypes should also be tested in secondary validation assays, that do not need to be high content-based (depending on the number of hits). Moreover, dose response curves should be performed, looking for compounds with low half maximal effective concentrations. The mechanism of action of the compounds might give insights into disease pathophysiology and should be investigated, if unknown. Blood-brain-barrier permeability is also important to determine when considering the most effective delivery route for central nervous system-acting drugs. Finally, further steps towards clinical translation should include other preclinical models and/ or drug repurposing studies, as appropriate.

## Declaration of Competing Interest

The authors declare that they have no known competing financial interests or personal relationships that could have appeared to influence the work reported in this paper.

The authors declare the following financial interests/personal relationships which may be considered as potential competing interests:

Robin Ketteler reports financial support was provided by Medical Research Council. Apostolos Papandreou reports financial support was provided by National Institute for Health Research. Manju A Kurian reports financial support was provided by Sir Jules Thorn Charitable Trust. Manju A Kurian reports financial support was provided by National Institute for Health Research. Apostolos Papandreou reports financial support was provided by Action Medical Research. Manju A Kurian reports financial support was provided by Wellcome Trust. Robin Ketteler reports financial support was provided by University of Pennsylvania. Manju A Kurian reports financial support was provided by Rosetrees Trust. Apostolos Papandreou, Serena Barral, Manju A Kurian reports a relationship with NIHR Great Ormond Street Hospital Biomedical Research Centre that includes: non-financial support. This research was supported by the National Institute for Health Research Biomedical Research Centre at Great Ormond Street Hospital for Children NHS Foundation Trust and University College London.
